# Diagnostic confusion, clinical chaos - an acute and transient psychotic disorder case report and brief historical review

**DOI:** 10.1192/j.eurpsy.2021.1392

**Published:** 2021-08-13

**Authors:** M. Carneiro, M. Piçarra, M. Reis, S. Neves

**Affiliations:** 1 Psychiatry, Centro Hospitalar e Universitario de Coimbra, Coimbra, Portugal; 2 General Practice, Unidade de Saude Familiar Topazio, Coimbra, Portugal

**Keywords:** Acute Transient Psychotic Disorder, Bouffee Delirante, historical review, Cycloid psychosis

## Abstract

**Introduction:**

Acute and Transient Psychotic Disorder (ATPD) is a group of rare psychotic disorders characterized by acute onset, symptom fluctuation and short duration typically followed by complete recovery. Throughout the time, there have been different attempts to classify these disorders (Bouffée Délirante, Cycloid Psychosis, etc.); nevertheless, in the current date, ATPD encompasses a broad spectrum of heterogenous clinical presentations with low diagnostic stability over time.

**Objectives:**

To describe a case of ATPD, highlighting the variability of its’ psychopathological phenomena and establishing a comparison with historical descriptions of this nosological entity.

**Methods:**

Clinical case report and brief review of literature.

**Results:**

V, 20-year old male without psychiatric history, presents in the emergency room exhibiting fluctuant psychopathology over the preceding two weeks – initially with depressive mood, anhedonia, apathy, bizarre behaviors and soliloquies; afterwards, showing paranoid delusional ideation; total insomnia in the previous 2-3 days; finally, showing grandiose delusional ideation; and throughout the episode, revealing pseudohallucinatory verbal activity assuming multiple identities. Several stress factors were identified in close time-relation with the onset of these symptoms. V. started risperidone 2mg 2id and quetiapine 100mg id and was discharged 2 weeks later, fully recovering from these psychopathological phenomena. V. remains asymptomatic at 6 months of follow-up.

**Conclusions:**

Historically, some classifications of this disorder focus on etiological factors, others on clinical evolution and course, and yet another group on separation from the Kraepelinian duality (schizophrenia and bipolar disorder). ATPD is a diagnosis with high clinical heterogeneity and low stability over time, which can have implications in follow-up and long-term outcome.

